# Mismatch repair genes in Lynch syndrome: a review

**DOI:** 10.1590/S1516-31802009000100010

**Published:** 2009-05-11

**Authors:** Felipe Cavalcanti Carneiro da Silva, Mev Dominguez Valentin, Fábio de Oliveira Ferreira, Dirce Maria Carraro, Benedito Mauro Rossi

**Affiliations:** 1 MSc. Doctoral student, Hospital AC Camargo, São Paulo, Brazil.; 2 MD, PhD. Surgeon at Hospital AC Camargo and researcher in the Research Center of Hospital AC Camargo, São Paulo, Brazil.; 3 PhD. Researcher in the Research Center of Hospital AC Camargo, São Paulo, Brazil.

**Keywords:** Lynch syndrome, Hereditary nonpolyposis colorectal cancer, DNA repair, Mutation, Cancer., Síndrome de Lynch, Câncer colorretal hereditário sem polipose, Reparo de DNA, Mutação, Câncer.

## Abstract

Lynch syndrome represents 1-7% of all cases of colorectal cancer and is an autosomal-dominant inherited cancer predisposition syndrome caused by germline mutations in deoxyribonucleic acid (DNA) mismatch repair genes. Since the discovery of the major human genes with DNA mismatch repair function, mutations in five of them have been correlated with susceptibility to Lynch syndrome: mutS homolog 2 (*MSH2*); mutL homolog 1 (*MLH1*); mutS homolog 6 (*MSH6*); postmeiotic segregation increased 2 (*PMS2*); and postmeiotic segregation increased 1 (*PMS1*). It has been proposed that one additional mismatch repair gene, mutL homolog 3 (*MLH3*), also plays a role in Lynch syndrome predisposition, but the clinical significance of mutations in this gene is less clear. According to the InSiGHT database (International Society for Gastrointestinal Hereditary Tumors), approximately 500 different LS-associated mismatch repair gene mutations are known, primarily involving *MLH1* (50%) and *MSH2* (40%), while others account for 10%. Much progress has been made in understanding the molecular basis of Lynch Syndrome. Molecular characterization will be the most accurate way of defining Lynch syndrome and will provide predictive information of greater accuracy regarding the risks of colon and extracolonic cancer and enable optimal cancer surveillance regimens.

## RESUMO

A síndrome de Lynch representa de 1-7% de todos os casos de câncer colorretal. É uma síndrome de herança autossômica dominante que predispõe ao câncer e é causada por mutações nos genes de reparo de ácido desoxirribonucléico (DNA). Desde a descoberta dos principais genes com função de reparo de DNA, mutações nos genes *MSH2*, *MLH1*, *MSH6*, *PMS2* e *PMS1* estão relacionadas com a susceptibilidade à síndrome de Lynch. Outro gene, *MLH3*, tem sido proposto como tendo papel na predisposição à síndrome de Lynch, porém mutações de significância clínica nesse gene não são claras. De acordo com o banco de dados InSiGHT (International Society for Gastrointestinal Hereditary Tumors), aproximadamente 500 diferentes mutações associadas à síndrome de Lynch são conhecidas, envolvendo primeiramente *MLH1* (50%), *MSH2* (40%) e outros (10%). Grandes progressos têm ocorrido para nosso entendimento das bases moleculares da síndrome de Lynch. A caracterização molecular será a forma mais precisa para definirmos a síndrome de Lynch e irá fornecer informações preditivas mais precisas sobre o risco de câncer colorretal e extra-colônico, além de permitir regimes otimizados de manejo.

## INTRODUCTION

Lynch syndrome represents 1-7% of all cases of colorectal cancer. It is an autosomal-dominant syndrome with high penetrance (about 85%), characterized by an accelerated process of carcinogenesis due to mismatch repair gene mutations.^1^^,^^2^^,^^3^ Lynch syndrome I (hereditary site-specific nonpolyposis colonic cancer, LSI) is characterized by inherited susceptibility to nonpolyposis colorectal carcinoma with an early age of onset, predilection for the proximal colon and multiple primary colorectal cancer. Lynch syndrome II (cancer family syndrome, LSII) has these features, but it is also associated with extracolonic cancer, particularly endometrial carcinoma (EC).^4^^,^^5^

**Chart 1.**
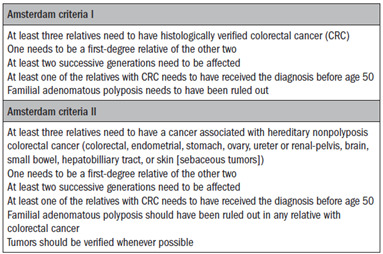
Amsterdam criteria I and II

Vasen et al. reported on the efforts of the International Collaborative Group on hereditary nonpolyposis colorectal cancer (ICG-HNPCC) and the establishment of a set of selection criteria for families with Lynch syndrome (Amsterdam Criteria I):^6^ 1) at least three relatives need to have histologically verified colorectal cancer; 2) one needs to be a first-degree relative of the other two; 3) at least two successive generations need to be affected; 4) at least one of the relatives with colorectal cancer needs to have received the diagnosis before the age of 50 years; and 5) familial adenomatous polyposis needs to have been ruled out. Different primary sites have been described in families with a possible diagnosis of Lynch syndrome: endometrium, stomach, ovaries, small bowel, ureter, renal pelvis, brain and hepatobiliary tract. Among the tumors at these sites, endometrial, ureteral, renal pelvic and small bowel cancers present the highest relative risk, and are therefore the most specific for Lynch syndrome. At the 1998 meeting, agreement was reached that these extracolonic tumors should be included, and a set of new clinical criteria was then proposed (Amsterdam Criteria II, ACII)^7^ (Chart 1).

The major genes involved are mutL homolog 1 (*MLH1*), mutS homolog 2 (*MSH2*), postmeiotic segregation increased 1 (*PMS1*), postmeiotic segregation increased 2 (*PMS2*), mutS homolog 6 (*MSH6*) and mutL homolog 3 (*MLH3*).^7^^,^^8^ Germline abnormalities in *MLH1* and *MSH2* genes are found in more than 90% of HNPCC mutation carriers.^9^ According to Papp et al., there are more than 500 different pathogenic mutations: 50% relating to *MLH1*, 40% to *MSH2* and 10% distributed among the others.^10^

Lynch syndrome individuals have an adenoma-carcinoma ratio of 1:1, while in the general population, it is 30:1. It is believed that all of the untreated polyps in mutation carriers will suffer malignant transformation, such that the risk of colorectal cancer is from 60% to 70% at the age of 70 years and 80% at 85 years.^11^^,^^12^

## HISTORY OF MISMATCH REPAIR GENES

The primary function of the DNA repair system is to eliminate the mismatch of base-base insertions and deletions that appear as a consequence of DNA polymerase errors during deoxyribonucleic acid (DNA) synthesis. Mismatch repair genes present several functions relating to genetic stabilization, such as correcting errors in DNA synthesis (Figure 1), ensuring fidelity of genetic recombination or participating in the initial steps of apoptotic responses to different classes of DNA damage.

The first studies on mismatch repair genes were developed in *Escherichia coli* (*E. coli*), in which the repair of the mismatch is characterized by absence of adenine methylation at the d(GATC) site within the newly synthesized DNA. Thus, the function of the hemimethylated d(GATC) strand signal in *E. coli* mismatch repair is to provide a nick on the unmethylated strand, which serves as the actual signal that directs the reaction.^13^ The sign that directs the correction of the replication error in eukaryotes still has not been defined but, similarly, a strand-specific cut or nick is sufficient to address the repair in extracts of mammal cells. This discovery, coupled with the observation that mismatch repair is more efficient on the lagging strand at the replication fork, suggests that the DNA termini that occur as natural intermediates during the replication (3’ terminus on the leading strand; 3’ and 5’ termini on the lagging strand) may suffice as strand signals to direct the correction of DNA biosynthetic errors in eukaryotic cells.^14^

The homo-oligomers responsible for initiating repairs on mismatches in *E. coli* are MutS and MutL. MutS is responsible for recognizing the mismatch and recruiting MutL to the mismatch location, thus starting the downstream activities.^14^^,^^15^^,^^16^ Mammal cells possess activity by two homologous MutS that work as heterodimers, in which MutSa is formed by the *MSH2*-*MSH6* complex and MutSb by *MSH2*-*MSH3*.^16^ Peltomaki^15^ identified six homologues to MutS in eukaryotic cells.

The *MSH2*-*MSH6* complex represents 80% to 90% of the cellular level of *MSH2* and its function is to recognize the mismatch of base-base insertions and deletions, to contain one or two unpaired nucleotides, but it is also capable of recognizing large deletions and insertions (Figure 1).^17^ It is believed that the protein *MSH6* is the subunit responsible for recognizing the mismatch.^18^

The *MSH2*-*MSH3* complex is responsible for recognizing and repairing insertions and deletions from two to eight nucleotides (Figure 1). Experimental studies have not demonstrated any association with Lynch syndrome,^19^^,^^20^ although the *MSH3* gene presents frequent somatic mutations in tumors and its inactivation can increase the potential consequences of mutations in other mismatch repair genes.

Eukaryotic cells also present three complex homologues to MutL of *E. coli*: *MLH1*-*PMS2* (MutLa), *MLH1*-*PMS1* (MutLb) and *MLH1*-*MLH3* (MutLg). MutLa is the most active of these complexes in humans and supports repairs initiated by the MutS complex.^13^ The MutLb complex has already been isolated; however, its involvement in DNA repair has not been demonstrated. Nor has the involvement of *MLH3* in the development of colorectal tumors.^21^

**Figure 1.4:**
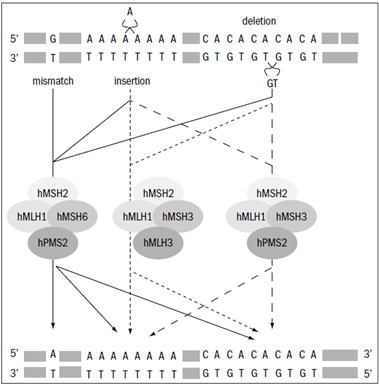
A model for mismatch repair. The top strand of the heteroduplex contains three anomalies that are resolved by the mismatch repair gene system: a base-base mispair, a single-nucleotide insertion loop in the top (primer) strand and a two-nucleotide deletion loop in the bottom (template) strand. All three are targeted by the *MSH2*/*MSH6*−*MLH1*/*PMS2* repair complex to give the product shown at the bottom of the figure. In all cases, the repair process is directed by a strand discontinuity (a nick) on the primer strand. The guanine-thymine mispair is corrected to adenine-thymine; the adenine stretch was shortened by one repeat unit and the sequence of cytosine-adenine dinucleotide repeats was extended by one. In the absence of *MSH6*, the two insertions/deletions are targeted by the *MSH2*/MSH3-*MLH1*/*PMS2* complex. The putative *MSH2*/MSH3-*MLH1*/*MLH3* complex is predicted (from yeast studies) to address a subset of insertion/deletion. Figure adapted from Jiricny.^17^

## MISMATCH REPAIR GENES IN LYNCH SYNDROME

The clinical understanding of Lynch syndrome has been complemented by the great progress in molecular genetics. The first molecular characterization of these tumors was at the beginning of the 1990s, with the characterization of microsatellite instability.^22^ Patients with Lynch syndrome were found to present variations in the number of repetitions in microsatellite units, caused by the flow in the correction system of mismatch repair genes.

For Lynch syndrome to be defined molecularly, a defect inherited in the mismatch repair genes needs to be demonstrated. Thus, germline mutations in at least one of the repair genes can be found in more than 80% of the individuals with Lynch syndrome.^23^ The *MLH1* gene (MIM#120436) is located in chromosome 3p21, with nineteen exons of 57.36 Kb. Today, more than 250 different germline mutations have been identified and *MLH1* is the most important gene in this syndrome.^24^ Several researchers have identified mutations in this gene, and its presence varies with the geographical area. In Lynch syndrome families reported in European and North American countries, stomach cancer is uncommon. In Asian countries such as Japan and Korea,^25^ and in Brazil,^26^ however, very high incidence of stomach carcinoma has been reported. Unfortunately, there are no studies in these countries showing the relative risk of developing stomach cancer in people with mutated mismatch repair gene.

The main types of mutations found in these genes are missense, nonsense, frameshift and splice junction mutations, which can easily be detected by automatic sequencing.^26^^,^^27^

The *MSH2* gene (MIM#120435) is in chromosome 2p16, with sixteen exons of 80.10 Kb.^23^*MSH2* and *MLH1* are responsible, together, for more than 64% of the cases of germline mutations in HNPCC.^28^^,^^29^

The *MLH6* (MIM#600678) gene is in chromosome 2p15, with 10 exons. Recently, germline mutations in this gene were recognized as a frequent cause of atypical Lynch syndrome (i.e. not fulfilling the Amsterdam criteria). The first studies on mutations in the *MLH6* gene indicated that the clinical phenotype of affected families differed from that of individuals with the classical Lynch syndrome caused by mutations in *MLH1* and *MSH2*. Its penetrance seems to be lower, although endometrial cancer is probably the most important clinical manifestation in women who carry a mutation in *MLH6*. Moreover, mutations in *MLH6* have a low incidence of microsatellite instability and preferentially occur in mononucleotide sequences.^30^^,^^31^^,^^32^^,^^33^^,^^34^

The *PMS1*(MIM#600258) and *PMS2* (MIM#600259) genes are located in the chromosomes 2q31-q33 and 7p22, with 16 Kb and 15 exons, respectively. According to data in the literature, they account for 5% of all cases of Lynch syndrome.^35^ Although the *PMS2* gene is crucial in the repair system, mutations have rarely been reported in the etiology of the Lynch syndrome, or in Turcot syndrome (one of the variants of Lynch syndrome).^4^ Controversies exist regarding the mechanism through which *PMS2* and *PMS1* may act to predispose towards cancer.^35^

## ABNORMALITIES IN MISMATCH REPAIR GENES IN LYNCH SYNDROME

Rossi et al.^36^ evaluated 25 Brazilian families with suspicions of Lynch syndrome. They found that 10 of them (40%) had mutations in the *MLH1* and *MSH2* genes: eight in *MLH1* (80%) and two in *MSH2* (20%). There were five missense, one nonsense, three frameshift and one splice defect. Lynch et al.^37^ identified mutations in 18 out of 56 individuals (32.1%).

In 1994, Nicolaides et al.^38^ were the first to suggest what the principal role of the *PMS1* and *PMS2* genes in Lynch syndrome was. In two unrelated families, they identified an in-frame deletion and a nonsense mutation in *PMS1* and *PMS2*, respectively.

One year later, in 1995, Hamilton et al.^39^ mapped fourteen American and Canadian families with Turcot syndrome and found that one child presented a germline mutation that was heterozygous for *PMS2*, with wide-ranging microsatellite instability in their normal cells. In 1997, Miyaki et al.^40^ reported a missense mutation in *PMS2* in a child with Turcot syndrome whose family did not have any history of this syndrome, although the child’s father carried the same mutation, albeit without cancer.

In 2000, De Rosa et al.^41^ found two missense mutations in *PMS2* in a patient with Turcot syndrome without any family history. In this study, the patient was young and the mutations were inherited from both his father and his mother, who were heterozygous for *PMS2*. Interestingly, although both the parents and another five members of the family were heterozygous for one of the mutations, none of them presented any increased predisposition towards cancer. This discovery led the researchers to make the interpretation that biallelic inactivation of the *PMS2* gene would be needed in order to produce predisposition towards cancer, thereby confirming the idea of the two-hit model proposed by Knudson.^42^

More recently, Nakagawa et al.^35^ found another seven mutations on the *PMS2* gene, through discovering a gene homologous to *PMS2*, also located in chromosome 7p22-23, very close to it. This gene was named *PMS2CL* and it presented 97% similarity with *PMS2*. This homologue has many similarities with the area in which the *PMS2* protein interacts with the *MLH1* protein to form the *MLH1*-*PMS2* complex. It is formed by the COOH terminus and is capable of generating false unmutated alleles (wild-type alleles). According to data from the National Center for Biotechnology Information, there are at least 13 sequences homologous to *PMS2*, in different areas. Nakagawa et al.^35^ also stated that the presence of the *PMS2CL* gene hinders the detection of frameshift mutations in automatic sequencing. Furthermore, they stated that the small number of mutations in exons 1 to 5 of *PMS2* may be due to the existence of other homologous areas. This suggests that mutations in *PMS2* may be much more common than hitherto thought. The great number of homologues may have greatly decreased the ability to detect mutations in *PMS2*.

A recent study by Hendriks et al.^43^ identified seven mutations in *PMS2*, including four genomic rearrangements and three point mutations. Among these seven individuals, six presented an autosomal-dominant inheritance pattern. This strongly indicates that heterozygous *PMS2* can predispose towards cancer, i.e. contrary to the findings of De Rosa et al.^41^

### Genomic rearrangements

With the discovery of multiplex ligation-dependent probe amplification (MLPA), it became possible to recognize, surprisingly, that a great proportion of the mutations resulting from genomic rearrangements of one or several exons deletions affected the *MLH1* and *MSH2* genes.^44^

Ainsworth et al.^45^ found a significant rate of genomic rearrangements in 10 out of 67 English individuals (15%), with five different types of rearrangements. Zhang et al.^46^ evaluated 16 related Swiss individuals with suspected Lynch syndrome who did not present germline mutations in the *MLH1* and *MSH2* genes, and they found that five of them (31%) had different genomic deletions.

According to Pistorius et al.,^47^ genomic rearrangements represent a significant proportion of all pathogenic mutations in mismatch repair genes in patients with Lynch syndrome. Many studies have demonstrated that genomic rearrangements represent 15% to 55% of all mutations in mismatch repair genes.^48^^,^^49^^,^^50^^,^^51^^,^^52^^,^^53^^,^^54^ Pistorius et al.^47^ found fourteen genomic rearrangements, in 85 individuals (16.5%), of which four were in *MLH1* and another ten in *MSH2*.

### Abnormalities in the mismatch repair gene promoter

Despite much research on genetic abnormalities in the coding regions of the mismatch repair genes in Lynch syndrome cases, the genetic abnormalities in the promoter of these genes have been poorly investigated. Recent studies have been defining and characterizing germline mutations in the central area of the promoters of these genes. Shin et al.^55^ evaluated 141 Korean patients with Lynch syndrome and found three new mutations in the promoter area of *MSH2*. These were all in individuals in whom no germline mutations had been detected. These mutations in the promoter significantly decreased its activity, thus affecting the initiation of the transcription site and the binding site of the transcriptional factor, and resulting in a new DNA-protein complex. These results are indicative that mutations in *MSH2* promoter are responsible for the initial tumor-forming process in a minority of Lynch syndrome cases.

### Epigenetic changes

Gazzoli et al.^56^ demonstrated that 60% to 90% of CpG islands are methylated in the cytosine residue in the human genome, although areas rich in CG without methylation are frequently associated with active genes. Epigenetic changes in the human genome may affect the cytosine as well as the chromatin structure.^57^^,^^58^ The cytosine residue can acquire a methyl group on its fifth carbon atom, and this occurs on the strand contrary to the palindromic sequence of CpG. In the great majority of expressed genes, the CpG islands are found in the promoter. When methylation takes place, the transcription factors cannot link at the promoter area, thereby inhibiting transcription, and consequently the gene is silenced.

Studies have been demonstrating that hypermethylation of *MLH1*, also known as epimutation, is not limited to neoplastic cells. In certain patients, hypermethylation of a single allele originates during germination and is distributed throughout the somatic cells.^56^^,^^57^^,^^58^^,^^59^^,^^60^

Although germline mutations in genes are transmitted faithfully across the generations in a Mendelian pattern, epimutations do not involve changes to the DNA sequence. They are relatively unstable, because of the process of epigenetic reprogramming in germline cells. Hitchins et al.^61^ evaluated 24 patients with colorectal or endometrial cancer that began before the age of 50 years, presenting microsatellite instability but without germline mutations in mismatch repair genes. They found that two patients presented a typical germline epimutation in *MLH1*, with all their somatic cells hemimethylated. A son of one of these patients presented partial methylation of *MLH1*; however, the analysis of his spermatozoids did not demonstrate any methylation profile in that gene. In the other patient, even though she had passed on her haplotype to one of her children, no evidence of methylation was found, probably due to reversion during gametogenesis. These results suggest that incomplete reversion of the epimutation occurred, with the possibility that the donated allele might have greater susceptibility towards undergoing subsequent somatic methylation in the next generation.

Hitchins et al.,^62^ in a previous study, evaluated 160 individuals with a suspicion of Lynch syndrome. They found that one female patient presented hemimethylation in *MLH1*, and that allele was inherited from her mother. Thus, these results make it possible that epigenetic error may appear more frequently during oogenesis or may be maintained more strongly during this process. This hypothesis could be confirmed by the reversion of the epimutation in the patient’s son. However, paternal inheritance cannot be ruled out, considering that Suter et al.^60^ found the presence of methylation in the spermatozoids of one son of a carrier patient, albeit in a low proportion (< 1%).

So far, those observations suggest that hypermethylation associated with silencing of *MLH1* represents an alternative to the two-hit model.^58^^,^^63^

## GENOTYPE-PHENOTYPE CORRELATION

Even in the absence of typical clinical characterization, there need to be criteria for directing the search for inherited conditions. Hence, correct interpretation of the spectrum of extracolonic tumors in Lynch syndrome cases acquires great importance. The frequent new molecular findings have been of great importance for better defining the genotype-phenotype correlation and thus the spectrum of extracolonic tumors in such cases.

For Lynch syndrome cases, many molecular tools are available for investigating genetic changes. Detection of microsatellite instability is one of the most used techniques among colorectal cancer patients. Patients with microsatellite instability indirectly show abnormalities in mismatch repair genes that cause protein deficiencies and loss of repair. Detection of germline mutations by DNA sequencing is the gold standard method but this is expensive and can be replaced by dHPLC as prior screening for mutations because of its lower cost. The disadvantage of dHPLC is that it cannot detect the correct position of the mutation and, therefore, subsequent use of DNA sequencing may be necessary. In centers without advanced molecular laboratories, diagnosis and research on colorectal cancer cases routinely relies on interpretation of protein expression detected by immunohistochemistry. This technique may be very useful for detecting loss of protein expression from mismatch repair genes, although missense mutations may be very difficult to detect, because some of this mutations do not cause loss of expression, but can affect function.

A comparison between families with germline mutations in *MLH1* or *MSH2* and families with mutations in *MLH6* showed that the latter presented later onset of colorectal cancer (at the age of 54 years, versus 44 years for the MLH1 and MSH2 cases). The women in the *MLH6* group presented a low risk of developing colorectal cancer (30% up to the age of 71 years), but a high risk of developing endometrial cancer (71% up to the age of 71 years).^33^^,^^64^

Other studies have demonstrated that individuals with mutations in *MSH2* have a higher risk of developing cancer in the urinary tract, stomach and ovaries, compared with individuals with mutations in *MLH1*. Beck et al.^65^ suggested that families with cancer that did not fulfill the requirements of the Amsterdam criteria, but that carried germline mutations, mostly presented missense mutations that resulted in less severe structural changes to the coded proteins, thereby reducing the aggressiveness of the disease.

## CONCLUSION

Great progress has been achieved over the last few years with regard to better molecular characterization of Lynch syndrome. Together with continuous improvement in study methods, this has been supplying a great variety of tumor marker candidates of clinical importance. Therefore, genetic tests for germline mutations in mismatch repair genes not only make it possible to identify families with Lynch syndrome, but also are of great importance for surveillance and management of high-risk patients.
